# Diabetic cardiomyopathy and COVID-19: intersecting pathways and amplified cardiovascular risk

**DOI:** 10.3389/fphar.2025.1683159

**Published:** 2025-10-13

**Authors:** Swati Prakash, Priyanka Choudhury, Shradha Bisht

**Affiliations:** ^1^ Amity Institute of Pharmacy, Amity University Uttar Pradesh, Lucknow Campus, Lucknow, India; ^2^ Cell Biology, Neurobiology and Anatomy, Medical College of Wisconsin, Milwaukee, WI, United States; ^3^ Uttaranchal Institute of Pharmaceutical Sciences (UIPS), Uttaranchal University, Dehradun, Uttarakhand, India

**Keywords:** diabetic cardiomyopathy, COVID-19, endothelial dysfunction, cytokine storm, renin-angiotensin-aldosterone system, inflammation

## Abstract

Diabetic cardiomyopathy (DCM) is a diabetes-induced heart condition characterized by ventricular dysfunction without other cardiac diseases. Chronic hyperglycemia, insulin resistance, and metabolic disturbances drive myocardial damage through renin–angiotensin–aldosterone system (RAAS) activation, oxidative stress, mitochondrial dysfunction, advanced glycation end product (AGE) accumulation, and persistent inflammation. The COVID-19 pandemic, caused by SARS-CoV-2, has intensified cardiovascular risk in diabetic patients. The virus uses ACE2 receptors, abundant in the heart and other organs, enabling multi-organ injury. COVID-19 may worsen glucose control or induce new-onset diabetes via pancreatic injury, insulin resistance, and stress hyperglycemia. Pre-existing diabetes increases the risk of severe COVID-19, cytokine storms, endothelial dysfunction, and thrombosis. In combination, both conditions promote RAAS imbalance, exaggerated inflammation, and hypercoagulability, amplifying myocardial injury, fibrosis, and heart failure risk. This review highlights the intricate bidirectional relationship between DCM and COVID-19, emphasizing shared pathogenic mechanisms such as RAAS dysregulation, endothelial damage, cytokine overproduction, and coagulopathy. Understanding these overlapping pathways is crucial for developing effective preventive and therapeutic strategies to mitigate adverse outcomes in this vulnerable population.

## 1 Introduction

Diabetic cardiomyopathy (DCM) is a specific form of secondary cardiomyopathy directly linked to diabetes mellitus (DM). It is defined by major cardiovascular societies, including the American College of Cardiology, American Heart Association, and European Society of Cardiology, as ventricular dysfunction occurring in diabetic individuals without other common cardiac conditions like coronary artery disease or hypertension. Chronic diabetes progressively alters cardiac structure and function, predisposing patients to heart failure independent of traditional cardiovascular risk factors (Choudhury et al., 2024b; Radzioch et al., 2024; Salvatore et al., 2021). Long-term studies, such as the Framingham Heart Study, have shown that diabetes can raise the risk of heart failure up to fivefold, irrespective of sex. Insulin resistance (IR) and impaired glucose tolerance further amplify this risk, creating a cycle of metabolic dysfunction and cardiac decline (Salvatore et al., 2021).

The development of DCM involves complex molecular and cellular processes. Key drivers include myocardial energy imbalance, glucotoxicity, lipotoxicity, disrupted insulin signaling, mitochondrial dysfunction, endoplasmic reticulum (ER) stress, calcium dysregulation, oxidative stress, accumulation of advanced glycation end products (AGEs), and persistent metabolic stress. Collectively, these mechanisms impair cardiac structure and function, increasing vulnerability to heart failure (Jia et al., 2018).

The emergence of COVID-19, caused by severe acute respiratory syndrome coronavirus 2 (SARS-CoV-2), has added significant risk for individuals with diabetes and pre-existing cardiovascular conditions. Identified in Wuhan, China in late 2019 and declared a pandemic by March 2020, COVID-19 has become the most critical public health challenge of the 21st century, with over 778 million cases and more than 7 million deaths worldwide as of May 2025 (Chung et al., 2024; World Health Organization, 2024).

SARS-CoV-2 is an enveloped RNA virus in the Betacoronavirus genus, possessing the largest genome (∼30 kb) among RNA viruses. Its genome encodes structural, accessory, and non-structural proteins that enable infection and replication (Heshmati, 2023; Yuan et al., 2023). The spike (S) protein binds to angiotensin-converting enzyme 2 (ACE2) receptors on host cells, a process aided by host proteases such as transmembrane serine protease 2 (TMPRSS2) and cathepsin L, allowing viral entry, replication, and spread.

Because ACE2 is expressed in many tissues, including the lungs, heart, vasculature, and pancreas, COVID-19 can damage multiple organ systems. Infection triggers a robust inflammatory response with high levels of cytokines like interleukin-6 (IL-6) and tumor necrosis factor-alpha (TNF-α), driving endothelial dysfunction and widespread tissue injury. Clinical severity ranges from mild or asymptomatic cases to severe pneumonia, acute respiratory distress syndrome (ARDS), and death (Washirasaksiri et al., 2023).

In severe cases, COVID-19 worsens IR and promotes metabolic stress, both critical elements in DCM pathogenesis. SARS-CoV-2 infection also alters stress signaling pathways, contributing to hyperglycemia and disease progression (Steenblock et al., 2021). Evidence shows COVID-19 heightens cardiovascular risk, including stroke, arrhythmias, myocarditis, heart failure, and thromboembolism (Abbasi, 2022). The coexistence of diabetes and cardiovascular disease further magnifies the likelihood of critical illness and poor prognosis compared to either condition alone (Freaney et al., 2020; Hebbard et al., 2021). Given these heightened risks, understanding the molecular and cellular pathways linking diabetes, cardiovascular dysfunction, and COVID-19 is essential to unravel how SARS-CoV-2 exacerbates DCM and to guide targeted interventions.

Therefore, this review focuses on the intricate reciprocal relationship between DCM and COVID-19, emphasizing shared pathogenic mechanisms such as RAAS dysregulation, endothelial damage, cytokine overproduction, and coagulopathy. Comprehending these overlapping pathways is vital for devising effective preventive and therapeutic strategies to lessen adverse outcomes in the susceptible population.

## 2 Pathophysiology of diabetic cardiomyopathy

**FIGURE 1:**
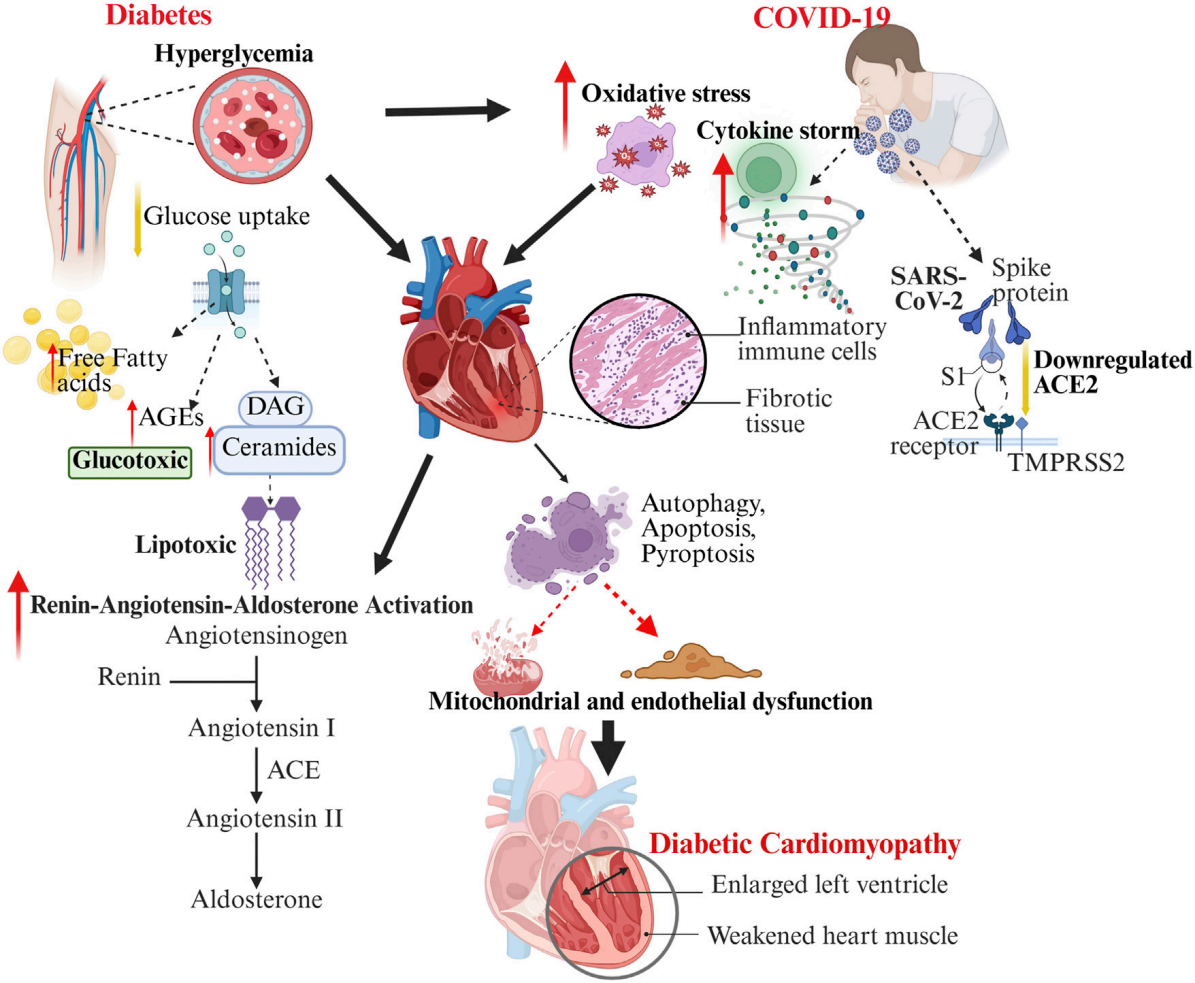
The schematic illustration depicting the intersecting molecular and cellular pathways linking diabetes and COVID-19 in the precipitation and exacerbation of DCM. Diabetes (left) leads to elevated blood sugar levels and disrupted lipid metabolism, resulting in glucotoxicity, lipotoxicity, oxidative stress, and activation of RAAS, which in turn causes mitochondrial and endothelial dysfunction. Conversely, COVID-19 (right) involves the entry of SARS-CoV-2 through ACE2, a reduction in ACE2 levels, the onset of a cytokine storm, oxidative stress, and the infiltration of inflammatory cells, all contributing to further cardiac damage. The interplay of these mechanisms disrupts autophagy, apoptosis, and fibrosis, ultimately heightening the risk and severity of diabetic cardiomyopathy, as evidenced by ventricular enlargement and impaired cardiac function.

DCM develops through a multifaceted interaction of molecular, cellular, and interstitial changes within the myocardium, driven by metabolic, structural, and signaling disruptions. Contributing factors include oxidative stress, metabolic disturbances, mitochondrial and endothelial dysfunction, inflammation, and dysregulation of pathways such as RAAS, AMP-activated protein kinase (AMPK), O linked N-Acetylglucosamine (O-GlcNAc), c-Jun N-terminal Kinase (JNK), Protein Kinase C (PKC), Mitogen-Activated Protein Kinase (MAPK), and Nuclear Factor Kappa-light-chain-enhancer of Activated B Cells (NFκB), along with ferroptosis. Together, these lead to structural remodeling—fibrosis, hypertrophy, microvascular dysfunction—and functional impairments in diastolic relaxation, compliance, and contractility (Figure 1) (Dhiman et al., 2024; Hu et al., 2017).

Under normal conditions, the adult heart predominantly relies on free fatty acids (FFAs) for energy but can also metabolize glucose, lactate, ketone bodies, and amino acids. In diabetes, metabolic inflexibility arises when glucose utilization declines due to impaired GLUT4 translocation to the sarcolemma, forcing increased FFA uptake. This shift promotes excessive fatty acid oxidation, which, although energy-producing, is less efficient, reduces ATP yield, and elevates reactive oxygen species (ROS) production while suppressing oxidative phosphorylation. Mitochondrial uncoupling further lowers cardiac energy efficiency by increasing oxygen consumption without proportionate ATP generation. These metabolic changes make the heart particularly vulnerable during ischemia, as glucose oxidation is compromised. Excess FFA availability exacerbates reliance on lipid metabolism, and imbalances between acylcarnitine synthesis and mitochondrial oxidation result in lipid intermediates such as diacylglycerols and ceramides. These molecules amplify oxidative stress, inflammation, and contractile dysfunction. Because cardiomyocytes lack the capacity to store excess lipids, FFA accumulation inflicts cellular injury, advancing DCM progression (Ho et al., 2022). Cluster of Differentiation 36 (CD36), a scavenger receptor responsible for long-chain FFA uptake, plays a central role in this process. Dysregulated CD36 distribution disrupts cardiac energy balance, while hyperglycemia and hyperlipidemia stimulate its translocation to the plasma membrane, further boosting FFA absorption (Shu et al., 2022).

AMPK, a master regulator of cellular energy homeostasis, is a key protective factor in metabolic disturbances underlying DCM. Normally, AMPK activation promotes energy balance by enhancing autophagy, reducing oxidative stress, mitigating inflammation and apoptosis, and improving insulin sensitivity and glucose uptake in cardiomyocytes (Entezari et al., 2022). However, in DCM, diabetes-associated metabolic and signaling defects impair AMPK activation. Hyperglycemia and hyperlipidemia disrupt upstream kinases (Liver Kinase B1: LKB1 and Calcium/Calmodulin-Dependent Protein Kinase Kinase β: CaMKKβ), decreasing AMPK phosphorylation. Concurrently, ROS, pro-inflammatory cytokines such as TNF-α and IL-6, mitochondrial dysfunction (lowering AMP/ATP ratios), reduced levels of FGF21 and adiponectin, post-translational modifications, and insulin resistance all converge to suppress AMPK activity (Zeng et al., 2024). This inhibition depletes cellular energy reserves, exacerbating cardiac dysfunction. Restoring AMPK activation has been shown to counteract these effects, preserving myocardial function and slowing DCM progression (Haye et al., 2020).

Blood glucose levels have a strong correlation with AGE accumulation in the body. Hyperglycemia accelerates non-enzymatic protein glycosylation, promoting AGE formation, particularly in the context of IR. AGEs interact with their receptor, receptor for AGEs (RAGE), activating downstream signaling cascades, including NFκB and PKC pathways, which drive ROS production, inflammation, and cardiac dysfunction. In DCM, hyperglycemia also facilitates AGE binding to Myeloid Differentiation Protein 2 (MD2) transporters, forming an AGE–MD2–TLR4 complex that activates pro-inflammatory pathways. Beyond inflammation, AGEs induce protein modifications, enhance collagen cross-linking, and accelerate atherosclerosis. Through the AGE/RAGE axis, fibroblasts transition into myofibroblasts, leading to extracellular matrix deposition and maladaptive cardiac remodeling (Cheng et al., 2023; Zeng et al., 2024). In addition to the above, NFκB activation further amplifies signaling by upregulating MAPK, PKB, and PPAR pathways. The resulting molecular responses promote inflammation, fibrosis, and hypertrophy, contributing to DCM progression.

Ferroptosis, a distinct form of regulated cell death dependent on iron but independent of apoptosis, also plays a role in diabetic hearts. It is characterized by lipid peroxide accumulation and excessive oxidative stress. In this setting, iron overload impairs antioxidant defenses, increases ROS production, and drives ferroptosis, leading to cardiomyocyte loss, fibrosis, and functional decline (Gawargi and Mishra, 2024; Tian et al., 2024).

## 3 Diabetes and COVID-19: increased severity, metabolic disruption, and cardiovascular complications

### 3.1 Impact of COVID-19 on glucose metabolism and diabetes development

Growing evidence indicates that SARS-CoV-2 infection not only worsens outcomes in individuals with pre-existing diabetes but may also precipitate new-onset diabetes in previously healthy individuals (Al-Aly et al., 2021). For instance, a study from the United Kingdom reported a significant increase in new cases of type 1 diabetes (T1D) among children aged 2–17 years during the pandemic (Unsworth et al., 2020). Similarly, research from China found that patients with COVID-19 exhibited higher rates of ketosis and diabetic ketoacidosis, regardless of whether they had a prior diabetes diagnosis (Li J. et al., 2020). A global meta-analysis further corroborates these findings, indicating that more than half of COVID-19 patients develop hyperglycemia, and approximately one-third may experience diabetic ketoacidosis (Thaweerat, 2020).

Mechanistically, several pathways link SARS-CoV-2 infection with disrupted glucose homeostasis. The virus can directly infect pancreatic tissue via ACE2 receptors, similar to the original SARS coronavirus, leading to local inflammation, β-cell dysfunction, and potentially autoimmune destruction of insulin-producing cells (Li et al., 2003; Thaweerat, 2020). In addition, viral infections may stimulate anti-pancreatic antibody production, initiating autoimmune responses that trigger T1D (Marchand et al., 2020; Rubino et al., 2020). Beyond direct pancreatic effects, COVID-19 induces a surge in stress hormones such as glucocorticoids and catecholamines, which exacerbate IR and hyperglycemia (Wang et al., 2020). This persistent metabolic disruption may also underline aspects of long COVID, such as prolonged fatigue and malaise.

### 3.2 Bidirectional risk: diabetes worsens COVID-19 severity

While COVID-19 can contribute to the onset or worsening of diabetes, the reverse is equally concerning: pre-existing diabetes significantly increases the severity and mortality risk of SARS-CoV-2 infection. Large cohort studies consistently show that diabetes is associated with greater likelihood of severe pneumonia, lymphopenia, prolonged hospitalization, and higher mortality rates (Guo et al., 2020; Petrilli et al., 2020). For example, data from New York indicated that diabetes, alongside obesity and hypertension, was highly prevalent among hospitalized COVID-19 patients and strongly correlated with mortality (Petrilli et al., 2020).

Poor glycemic control during COVID-19 treatment further compounds poor outcomes. A US cohort study found that mortality was nearly four times higher in patients with pre-existing diabetes or hyperglycemia compared to those with normal blood glucose levels (Bode et al., 2020). Alarmingly, even patients without prior diabetes who developed stress-induced hyperglycemia had significantly poorer prognosis (Li X. et al., 2020). Clinical studies have consistently reported higher rates of cardiovascular complications, including heart failure and arrhythmias, in COVID-19 patients with diabetes (Barron et al., 2020; Huang et al., 2020). Collectively, these findings highlight that while diabetes does not increase susceptibility to SARS-CoV-2 infection *per se*, it strongly predisposes patients to severe disease and complications (Apicella et al., 2020).

This interplay underscores that pre-existing diabetes not only worsens COVID-19 outcomes through hyperglycemia and immune dysregulation but also amplifies the cardiovascular complications associated with SARS-CoV-2 infection.”

## 4 Intersection of diabetic cardiomyopathy and COVID-19

### 4.1 Increased susceptibility to severe COVID-19 in patients with diabetes

Clinical evidence strongly supports diabetes as a major risk factor for severe COVID-19 outcomes. Both type 1 and type 2 diabetes confer markedly increased risks of hospitalization and severe disease, with adjusted odds ratios of ∼3-4 compared to non-diabetic individuals (Gęca et al., 2022; Gregory et al., 2021). A UK population study of over 23,000 COVID-19–related deaths reported that more than 30% of cases had diabetes—five times higher than population prevalence—with diabetic patients succumbing at younger ages (Barron et al., 2020). Importantly, poor glycemic control correlates with greater treatment requirements, prolonged hospitalizations, and higher mortality (Carey et al., 2018; Zhu et al., 2020). Moreover, coexisting cardiovascular disease and diabetes synergistically amplify the risk of complications, including heart failure (Freaney et al., 2020). Together, these findings highlight diabetes as a critical determinant of COVID-19 severity, underscoring the need to elucidate underlying mechanisms such as RAAS dysregulation, endothelial dysfunction, and heightened inflammatory signaling.

### 4.2 RAAS dysregulation in diabetes and COVID-19: ACE2 and furin expression

A central mechanism linking diabetes, COVID-19, and DCM is the dysregulation of the RAAS, specifically the imbalance between ACE/Angiotensin-II and ACE2/Angiotensin-(1–7) signaling. In diabetes, increased ACE activity elevates Angiotensin-II, through AT_1_ receptor activation, promotes oxidative stress, NF-κB–mediated inflammation, insulin resistance, and TGF-β–driven fibrosis (Hsueh and Wyne, 2011; Patel et al., 2016). These maladaptive processes impair cardiomyocyte metabolism, induce hypertrophy, and contribute to extracellular matrix remodeling and diastolic dysfunction.

Under normal conditions ACE2 generates Angiotensin-(1–7), which acts on the Mas receptor to exert anti-inflammatory, anti-fibrotic, and vasodilatory effects by inhibiting NOX activity, improving nitric oxide bioavailability, and attenuating TGF-β signaling (Batista et al., 2023; Offringa et al., 2020; Santos et al., 2019; Zhong et al., 2010). During COVID-19, SARS-CoV-2 binds ACE2 and triggers clathrin- and AP2-dependent endocytosis, followed by lysosomal degradation, leading to reduced membrane-bound ACE2 expression (Gheblawi et al., 2020; Jackson et al., 2022; Lu et al., 2022; South et al., 2020). This loss is especially harmful in tissues with high ACE expression, such as the lungs, promoting vasoconstriction, oxidative stress, fibrosis, and inflammation (Gheblawi et al., 2020). The resulting imbalance between Angiotensin II and Angiotensin-(1–7) exacerbates tissue injury, endothelial dysfunction, and cytokine activation, contributing to severe COVID-19 outcomes. Thus, ACE2’s dual role—as a viral entry receptor and a regulator of cardiovascular homeostasis—underscores its central importance in DCM progression in the context of COVID-19.

Building on this framework, an important question remains: how is ACE2 expression regulated across different tissues and pathological contexts in diabetes, and how might this variability influence both RAAS activity and viral susceptibility? Understanding these dynamics could inform precision strategies to mitigate COVID-19–related cardiac complications in diabetic patients.

#### 4.2.1 Tissue-specific ACE2 expression in diabetes

In diabetes, RAAS dysregulation alters ACE2 expression in a tissue- and context-dependent manner. In diabetic rodent models (e.g., streptozotocin, db/db, NOD), ACE2 activity or protein abundance is generally increased in lung, kidney, heart, and pancreas, often in parallel with larger increases in ACE, suggesting a compensatory shift in the ACE2:ACE ratio (Riera et al., 2014; Roca-Ho et al., 2017; Wysocki et al., 2006). By contrast, human data are more heterogeneous. Mendelian randomization supports a causal link between diabetes-related traits and higher lung ACE2 expression, but direct tissue-level confirmation is limited (Deravi et al., 2020; Xie et al., 2021). In human subcutaneous adipose tissue, ACE2 mRNA is consistently lower in type 2 diabetes and obesity (P ≈ 9 × 10^−6^), strongly correlating with adverse metabolic traits such as hyperinsulinemia, higher Body Mass Index, and dyslipidemia (El-Sayed Moustafa et al., 2022). However, epicardial adipose tissue may show higher ACE2 levels in the same context, underscoring depot-specific variation (Couselo-Seijas et al., 2021).

These discrepancies likely reflect tissue specificity, assay modality (mRNA, protein, enzymatic activity, soluble ACE2), biological context (membrane-bound vs. soluble, compensatory vs. pathogenic), and modifiers such as glycemic control, medications, inflammation, and cell-type composition.

Taken together, ACE2 regulation in diabetes is heterogeneous across tissues, species, and experimental systems. Future studies should (i) clearly define tissue source and assay modality, (ii) distinguish membrane-bound from soluble ACE2, (iii) determine whether observed changes are compensatory or pathogenic, and (iv) integrate tissue-level, genetic, and functional data to better define how RAAS dysregulation links diabetes to both SARS-CoV-2 susceptibility and DCM.

#### 4.2.2 Furin and TMPRSS2: facilitators of viral entry

SARS-CoV-2 entry requires not only ACE2 binding but also host protease–mediated priming of the viral spike protein. TMPRSS2 and furin are the key proteases that cleave the spike protein, enabling membrane fusion and cellular entry (Muniyappa and Gubbi, 2020). In diabetes, both ACE2 and furin are upregulated, thereby enhancing viral infectivity and replication (Muniyappa and Gubbi, 2020). Elevated furin levels are also independently associated with diabetes progression and increased mortality risk (Adu-Agyeiwaah et al., 2020). Collectively, these observations suggest that diabetic patients may harbor a cellular environment particularly permissive to SARS-CoV-2 entry and propagation.

#### 4.2.3 Evidence of cardiac ACE2 dysregulation in diabetics with COVID-19

ACE2 is expressed in more than 7.5% of cardiomyocytes, providing a direct route for SARS-CoV-2 invasion into heart tissue (Zou et al., 2020). Viral RNA has been detected in cardiac samples by RT-PCR, confirming myocardial infection (Yao et al., 2020). Recent autopsy studies have shown markedly elevated ACE2 protein expression in cardiomyocytes of diabetic patients, with distinctive peri-nuclear localization and enrichment of glycosylated ACE2 isoforms (D’Onofrio et al., 2021). Immunoblot analyses further demonstrated increased levels of both glycosylated ACE2 and TMPRSS2 in diabetic myocardium compared to non-diabetic controls. These findings suggest that diabetes not only increases ACE2 expression but also modifies its glycosylation status, collectively facilitating enhanced viral binding, entry, and replication (D’Onofrio et al., 2021).

Such cardiac ACE2 upregulation may increase viral entry points, leading to higher viral loads, direct cytopathic effects, viral myocarditis, myocardial edema, and progressive cardiac dysfunction in diabetic patients with COVID-19.

#### 4.2.4 Dual impact of hyperglycemia on ACE2

Hyperglycemia exerts a bidirectional influence on ACE2 expression. Acute hyperglycemia may transiently increase ACE2 levels at the cell surface, thereby facilitating viral attachment and entry. Conversely, chronic hyperglycemia appears to downregulate ACE2, leading to unopposed Angiotensin II signaling, greater oxidative stress, and heightened inflammation (Roca-Ho et al., 2017; Yang et al., 2006).

Importantly, ACE2 is expressed not only in cardiovascular tissue but also in pancreatic β-cells, raising the possibility that SARS-CoV-2 directly damages the endocrine pancreas, contributing to new-onset diabetes or worsening of pre-existing disease (Roca-Ho et al., 2017; Yang et al., 2006).

Thus, ACE2 upregulation in diabetic hearts may confer cardioprotection under basal conditions but paradoxically increases vulnerability to severe COVID-19 by enhancing viral entry and intensifying RAAS imbalance after infection. This complex interplay underscores the need for therapeutic strategies that can selectively modulate ACE2 function. Potential approaches include RAAS inhibitors, recombinant ACE2 therapy, and Angiotensin-(1–7) agonists, which may restore protective RAAS signaling while mitigating viral-mediated myocardial injury (Verdecchia et al., 2020).

### 4.3 Endothelial dysfunction in diabetes and COVID-19

#### 4.3.1 Impact of diabetes on the endothelium

In DM, chronic hyperglycemia and IR disrupt endothelial homeostasis through multiple interrelated mechanisms. Sustained elevations in blood glucose activate protein kinase C and increase metabolic flux through the sorbitol and pentose phosphate pathways. Together, these changes drive excess production of ROS and exacerbating oxidative stress (Calles-Escandon and Cipolla, 2001; Endemann and Schiffrin, 2004). Elevated ROS levels, in turn, reduce the bioavailability of NO, a key vasodilator, and promote endothelial cell apoptosis, ultimately impairing vascular function.

A systemic reduction in NO bioavailability is a hallmark of diabetes, affecting both macrovascular and microvascular beds. This deficit arises not only from increased ROS but also from reduced expression and activity of endothelial nitric oxide synthase (eNOS). Experimental studies consistently report decreased eNOS expression in various organs of diabetic rodent models, including the heart (Chiu et al., 2016; Peng et al., 2015; Tsukahara et al., 2015), brain (Poittevin et al., 2015), kidneys (Advani et al., 2013), and retina (Omae et al., 2013). Importantly, restoration of NO levels—either via endogenous pathways or exogenous supplementation—has been shown to mitigate DCM (Baumgardt et al., 2016).

In addition to reduced expression, diabetes alters eNOS localization and post-translational regulation. Despite preserved mRNA and total protein levels, mislocalization of eNOS within cardiac endothelial cells has been documented, suggesting impaired trafficking or activation that limits NO synthesis. These molecular alterations closely correlate with cardiac dysfunction in diabetic rats and in animals treated with L-NAME, a NOS inhibitor, highlighting the pivotal role of NO signaling in DCM (Sampaio et al., 2002). Furthermore, a reciprocal relationship exists between endothelial dysfunction and IR. Under normal conditions, insulin signaling promotes vasodilation and exerts anti-inflammatory effects. However, in insulin-resistant states, chronic hyperinsulinemia paradoxically induces oxidative stress and vascular inflammation, thereby worsening endothelial impairment (Calles-Escandon and Cipolla, 2001).

Taken together, these pathophysiological mechanisms converge to produce marked endothelial dysfunction in diabetes. Key hallmarks include diminished vasodilatory capacity, increased vascular permeability, a persistent proinflammatory milieu, and heightened prothrombotic activity. Collectively, these changes accelerate the progression of diabetes-associated cardiovascular complications (Le Brocq et al., 2008; Shaw et al., 2014).

#### 4.3.2 Impact of COVID-19 on the endothelium

SARS-CoV-2 infection further exacerbates endothelial dysfunction, adding to the vascular injury already observed in diabetes and related conditions. The virus gains cellular entry through the ACE2 receptor, which is abundantly expressed on endothelial cells (Varga et al., 2020). Post-mortem and histological studies have confirmed the presence of SARS-CoV-2 in endothelial cells of the lung and other organs, accompanied by evidence of inflammation, endothelialitis, and microvascular thrombosis (Ackermann et al., 2020; Varga et al., 2020).

Once inside the endothelium, SARS-CoV-2 directly induces cell injury and apoptosis, compromising both the antithrombotic and barrier functions of the vascular lining. This disruption triggers a cascade of maladaptive responses: excessive platelet activation, upregulation of adhesion molecules (e.g., Intercellular Adhesion Molecule 1 (ICAM-1), Vascular Cell Adhesion Molecule 1 (VCAM-1)), and a shift toward a hypercoagulable state. Together, these changes culminate in widespread microvascular dysfunction and multi-organ injury (Varga et al., 2020). Supporting these pathological observations, clinical studies report elevated circulating levels of soluble ICAM-1, VCAM-1, and other markers of endothelial activation in COVID-19 patients, correlating with disease severity.

A key unresolved question is whether endothelial injury in COVID-19 results primarily from direct viral infection or indirect systemic effects, such as cytokine-driven inflammation and coagulopathy. While viral particles have been identified within endothelial cells, some studies argue that systemic inflammation, cytokine storm, and coagulopathy may play a more dominant role than direct cytopathic effects (Basta, 2021; Lui et al., 2023). This distinction is clinically important, as it influences whether antiviral strategies, endothelial-protective agents, or anti-inflammatory therapies should be prioritized in treatment.

### 4.4 Inflammation in diabetes and COVID-19

#### 4.4.1 Cytokine storm and hyperinflammation

Severe COVID-19 is characterized by pronounced systemic inflammation, as evidenced by elevated levels of C-reactive protein (CRP), ferritin, pro-inflammatory cytokines, and a high neutrophil-to-lymphocyte ratio (Henry et al., 2020; Petrilli et al., 2020; Zhou et al., 2020). Consistently, autopsy studies have revealed widespread inflammatory infiltration across multiple organs, including lungs, heart, kidneys, spleen, and pancreas (Diao et al., 2021; Feng et al., 2020; Xu et al., 2020), highlighting the multi-organ impact of systemic inflammation and its interplay with metabolic disturbances.

The “cytokine storm” defined by uncontrolled release of mediators such as IL-1, IL-6, IL-8, IL-10, TNF-α, and IFN-γ is considered a key driver of rapid clinical deterioration (Behrens and Koretzky, 2017). Notably, elevated IL-6 and lactate dehydrogenase (LDH) levels within 24 h of hospital admission have been identified as independent predictors of COVID-19 severity and poor outcomes (Zeng et al., 2020). However, whether the cytokine storm alone fully explains severe disease remains debated. Multiple studies indicate that cytokine levels in COVID-19—particularly IL-6—are substantially lower than those observed in classical cytokine release syndromes. For example, IL-6 concentrations in COVID-19 average around 36.7 pg/mL, compared with nearly 100-fold higher levels in CAR-T cell–induced cytokine release syndrome and even higher levels in sepsis and ARDS (Eljaaly et al., 2021; Leisman et al., 2020; Yu et al., 2021).

This discrepancy raises important questions about the relative contributions of different pathogenic mechanisms. While systemic inflammation is undoubtedly central, complementary processes—such as endothelial dysfunction, complement activation, and thromboinflammation—appear to play equally critical, if not primary, roles in driving organ injury and mortality. Histopathological studies support this complexity, revealing diffuse alveolar damage with hyaline membranes, extensive lung parenchymal inflammation, myocardial involvement, hepatic lymphocytic infiltration, cerebral macrophage clustering, glomerular microthrombi, and focal pancreatitis in fatal cases (Eketunde et al., 2020). Importantly, these observations do not fully distinguish between direct viral cytopathic effects and secondary immune-mediated damage, highlighting a key knowledge gap in understanding COVID-19 pathogenesis.

Severe COVID-19 frequently progresses to ARDS, characterized by severe alveolar edema, diffuse inflammation, and elevated markers such as CRP and erythrocyte sedimentation rate (Huang et al., 2020; Zhou et al., 2020). Concurrent increases in D-dimer and ferritin indicate a hypercoagulable state, linking systemic inflammation to both microvascular and macrovascular complications (Cheema et al., 2020). Critically, these findings emphasize that therapeutic strategies focused solely on cytokine inhibition may be insufficient. Effective interventions likely require a multi-pronged approach targeting viral replication, immune dysregulation, endothelial injury, and coagulopathy. Furthermore, additional research is needed to quantify the relative contribution of each mechanism across patient populations, identify biomarkers predicting which pathways dominate in individual patients, and determine how pre-existing comorbidities, such as diabetes and cardiovascular disease, modulate these interactions.

#### 4.4.2 Diabetes and exaggerated inflammatory response

Diabetes mellitus (DM) is characterized by chronic low-grade inflammation, with elevated baseline IL-1β and IL-6 (Rao et al., 2020). This pre-existing inflammatory milieu predisposes patients to exaggerated immune responses upon SARS-CoV-2 infection, increasing the risk of hyperinflammatory complications such as cytokine storm and multiorgan damage (Guo et al., 2020). However, it remains unclear whether this amplified response is due to intrinsic immune dysregulation in diabetes, to poor glycemic control during acute illness, or to the interaction of both.

Mechanistically, diabetes may impair viral clearance, enhance neutrophil activation, and promote neutrophil extracellular trap (NET) formation (Cicco et al., 2020). Yet, the clinical relevance of NETs in COVID-19 is still uncertain. Much of the evidence derives from small cohorts or *in vitro* studies, which may not fully capture *in vivo* dynamics. For instance, a longitudinal study of 93 hospitalized patients (201 blood samples) found that while markers such as cfDNA, MPO-DNA, Cit-H3, and NE-DNA were elevated, only cfDNA consistently correlated with disease severity and mortality—raising concerns that other markers might overestimate NET activity or reflect tissue damage rather than true NETosis mechanisms (de Diego et al., 2024). Similarly, several earlier investigations were limited to small sample sizes (e.g., 50 patients and 84 serum samples) or relied on laboratory-based assays where COVID-19 patient serum triggered NET release in control neutrophils—insights that are informative yet constrained by the artificial experimental conditions (Zuo et al., 2020).

Chronic hyperglycemia and IR further aggravate immune dysregulation through oxidative stress, impaired innate immunity, and activation of pathways such as AMPK/mTOR (Šestan et al., 2018). Excessive cytokine production, in turn, exacerbates IR by impairing glucose uptake in muscle and liver (Groop et al., 1989), creating a vicious cycle of hyperglycemia and inflammation. However, the contribution of this cycle to long-term outcomes is uncertain; it is debated whether it accelerates diabetic complications or merely reflects acute stress hyperglycemia.

Epidemiological evidence suggests that this inflammatory burden may influence both acute outcomes and the progression of diabetic complications. A nationwide French study linked pre-existing diabetic complications with higher COVID-19 mortality (Cariou et al., 2020), strengthens the association but does not establish causality. Additional longitudinal studies are required to clarify whether COVID-19 accelerates the trajectory of diabetic vascular complications or unmasks pre-existing subclinical disease.

Taken together, the convergence of oxidative stress, inflammation, and metabolic dysregulation provides a mechanistic basis for the exacerbation of vascular complications in diabetic patients with COVID-19.

### 4.5 Hypercoagulability and risk of thrombosis in DCM patients with COVID-19

#### 4.5.1 Thrombotic complications in COVID-19

Thrombotic events are a frequent and serious complication of severe COVID-19, occurring in up to half of ICU patients. Laboratory findings often include elevated D-dimer, prolonged clotting times, and disseminated intravascular coagulation, reflecting the combined effects of endothelial injury, systemic inflammation, platelet activation, and immobilization associated with critical illness (Moores et al., 2020; Yan et al., 2020). Viral infection promotes endothelial apoptosis, inflammation, and increased vascular permeability, creating a pro-thrombotic environment that predisposes patients to thromboembolism and stroke (Varga et al., 2020).

#### 4.5.2 Coagulation abnormalities in diabetes

DM is inherently a prothrombotic condition, driven by the interplay of chronic inflammation, endothelial dysfunction, platelet hyperactivity, and impaired fibrinolysis (Dunn and Grant, 2005). Chronic hyperglycemia and hyperinsulinemia increase the expression of plasminogen activator inhibitor-1 (PAI-1), suppressing fibrinolytic capacity and promoting persistent clot formation (Thögersen et al., 1998). In addition, hyperglycemia also promotes AGEs, which enhance tissue factor (TF) expression and activate the RAGE pathway, further stimulating coagulation and inflammation (Iwasaki et al., 2007; Manigrasso et al., 2014; Ramasamy et al., 2012; Rojas et al., 2021).

Hyperglycemia also damages the endothelial glycocalyx, exposing procoagulant surfaces and increasing platelet adhesion. Diabetic individuals frequently form denser fibrin clots with prolonged lysis times due to glycation of fibrinogen and other clotting proteins, further impairing clot resolution (Vaidyula et al., 2006). These baseline thrombotic vulnerabilities are exacerbated by acute hyperglycemia and glycemic variability, which amplify endothelial dysfunction and platelet hyperreactivity, increasing the risk of thromboembolic complications during COVID-19 (Nusca et al., 2019; Olesen et al., 2019; Overvad et al., 2015).

#### 4.5.3 Role of PAI-1, D-Dimer, and synergistic hypercoagulability in diabetes and COVID-19

High PAI-1 levels are a hallmark of DM and rise further during SARS-CoV-2 infection (Basurto et al., 2024). Elevated PAI-1 impairs tissue plasminogen activator (t-PA) activity, limiting fibrinolysis and promoting clot persistence. Adiposity, commonly present in DM, further exacerbates this prothrombotic environment by increasing secretion of IL-6, TNF-α, and IL-1β, which in turn upregulate PAI-1 expression (Steenblock et al., 2021).

D-dimer, a fibrin degradation product, serves as a robust prognostic marker in COVID-19. Levels above 2.0 μg/mL are strongly associated with higher in-hospital mortality (Zhang et al., 2020). Concordantly, patients with T2D or stress-induced hyperglycemia consistently exhibit higher D-dimer levels, reflecting increased clot formation and impaired fibrinolysis, which correlate with poorer clinical outcomes (Basurto et al., 2024). The combination of hyperglycemia, insulin resistance, and inflammation creates a synergistic environment that not only promotes clot formation but also impairs clot resolution, thereby worsening outcomes in diabetic patients with COVID-19 (Li et al., 2021).

Emerging experimental evidence highlights the therapeutic potential of targeting PAI-1. Pharmacological inhibition of PAI-1, for example, using PAItrap3, has been shown to improve glucose control and reduce thrombotic risk in diabetic models (Tang et al., 2020). These findings emphasize the intricate interplay between metabolic dysregulation and coagulopathy in patients with diabetes and COVID-19 and underscore the need for integrated therapeutic strategies that address both pathways simultaneously. However, the optimal timing, dosage, and clinical efficacy of PAI-1-targeted interventions in human COVID-19 patients, particularly those with diabetes, remain uncertain and warrant rigorous investigation through well-designed clinical trials.

## 5 Mechanistic overlap between DCM and COVID-19

DCM and COVID-19 share overlapping pathogenic mechanisms that exacerbate cardiac injury, including ACE2 downregulation, maladaptive RAAS activation, and heightened inflammation. In diabetes, baseline endothelial vulnerability, impaired NO signaling, and heightened thrombogenicity (Çalışkan et al., 2022; Drakos et al., 2021; Endemann and Schiffrin, 2004; Stockklauser-Färber et al., 2000) are further amplified by SARS-CoV-2–induced inflammation, oxidative stress, and coagulopathy (Gimbrone and García-Cardeña, 2016; Sandoo et al., 2010), creating a “double hit” that predisposes patients to severe cardiovascular complications, multiorgan injury, and higher mortality.

While this mechanistic convergence explains the increased risk of adverse outcomes in diabetic patients with COVID-19, much of the current evidence derives from animal models or post-mortem studies (Chung et al., 2025; Ma et al., 2022), and the precise pathways linking coronary microvascular impairment to clinical outcomes remain incompletely understood. These insights underscore the urgent need for integrated therapeutic strategies—targeting NO bioavailability, inflammation, and thrombosis—and for longitudinal studies and clinical trials to establish causality and optimize interventions in this high-risk population (Table 1).

**TABLE 1:** Mechanistic overlap between DCM and COVID-19.

Mechanism/Pathway	Role in DCM	Role in COVID-19	Overlap/Clinical implication	References
ACE2 signaling and RAAS dysregulation	Reduced ACE2 expression/activity shifts RAAS toward Ang II/AT1R signaling → vasoconstriction, hypertrophy, fibrosis	SARS-CoV-2 binding depletes ACE2, driving RAAS imbalance, Ang II–mediated inflammation, and vascular injury	Loss of ACE2 protective signaling is a shared mechanism; RAAS modulation (e.g., Angiotensin II receptor blockers, ACE2 mimetics) holds therapeutic promise	Gheblawi et al. (2020), Patel et al. (2016)
Inflammation/Cytokines	Chronic inflammation (↑ TNF-α, IL-6, IL-1β) drives remodeling and apoptosis	Cytokine storm with IL-6, IL-1β, TNF-α, IFN-γ surges causes systemic inflammation and cardiac damage	Inflammatory amplification worsens myocardial injury; IL-6 blockade or anti-inflammatory strategies may provide dual benefit	Bhaskar et al. (2020), Tsalamandris et al. (2019)
Endothelial dysfunction and microvascular injury	Hyperglycemia, AGEs, ROS → impaired NO bioavailability, vascular stiffness, microvascular rarefaction	SARS-CoV-2 infects endothelium (via ACE2), inducing endothelialitis, apoptosis, and microthrombosis	Pre-existing dysfunction amplifies COVID-19–induced vascular injury, raising risk of thromboembolism and ischemic complications	Chang et al. (2021), Islam et al. (2025)
Oxidative stress	Hyperglycemia/lipotoxicity → mitochondrial ROS, impaired contractility, apoptosis	Viral infection and hyperinflammation increase ROS, damaging cardiomyocytes and endothelium	Convergent ROS-mediated injury accelerates dysfunction; suggests role for antioxidant or mitochondrial-targeted therapies	Georgieva et al. (2023), Kaludercic and Di Lisa (2020), Tangos et al. (2022)
Fibrosis/ECM remodeling	TGF-β signaling and collagen deposition → myocardial stiffness, diastolic dysfunction	Post-COVID-19 pathology shows fibrosis, scar formation, persistent remodeling	Shared fibrotic pathways may lead to chronic HF in post-COVID diabetes; antifibrotic interventions are potential targets	Kamdar et al. (2024), Sun et al. (2025)
Metabolic dysregulation	Impaired glucose oxidation, excess fatty acid utilization, and lipotoxicity increase cardiac stress and inefficiency	SARS-CoV-2 alters host metabolism, worsens IR, and triggers stress hyperglycemia	Synergistic metabolic stress worsens outcomes; emphasizes importance of metabolic control and therapies (e.g., SGLT2 inhibitors)	Li et al. (2024), Panwar et al. (2025)
Hypercoagulability/Thrombotic risk	Diabetes is a prothrombotic state: ↑ PAI-1, platelet hyperactivity, impaired fibrinolysis, and dense fibrin clots	COVID-19 induces coagulopathy: elevated D-dimer, platelet activation, endothelial injury, microthrombosis, and increased venous thromboembolism risk	The combination of diabetes and COVID-19 synergistically increases thrombotic risk, predisposing to micro- and macrovascular events; highlights need for anticoagulation strategies	Altalhi et al. (2021), Chen et al. (2022), World Health Organization (2024)

## 6 Management strategies

**FIGURE 2:**
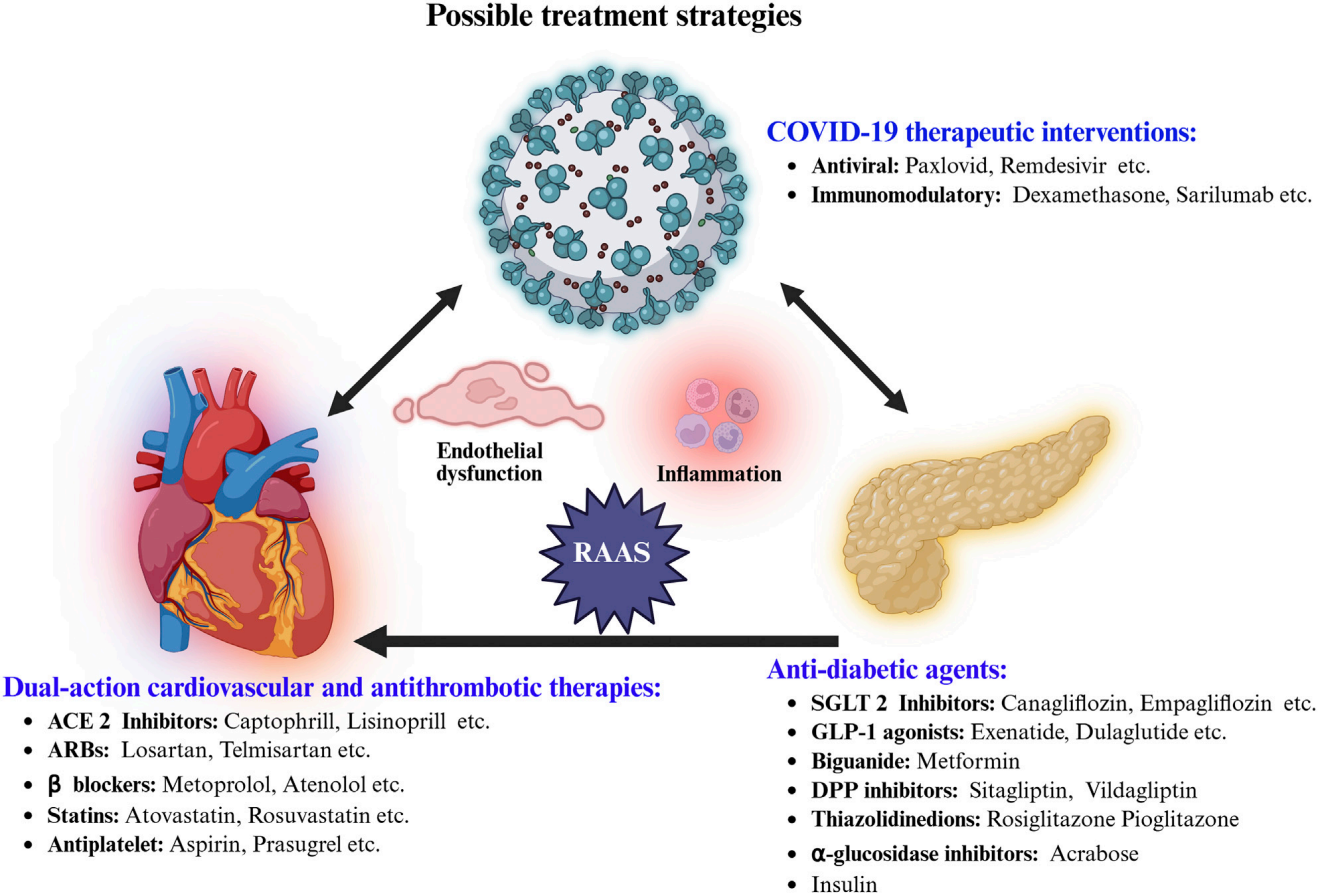
Integrated pharmacological strategies targeting COVID-19 complications in diabetes-highlighting anti-viral, immunomodulatory, anti-diabetic, cardioprotective, and anti-thrombotic agents aiming for improved treatment outcomes.

Patients with diabetes or DCM who contract COVID-19 face significantly higher risks of severe outcomes—including hospitalization, cardiovascular complications, and death—due to the convergence of metabolic dysregulation, endothelial injury, and systemic inflammation. Effective management requires a multidisciplinary approach that simultaneously addresses glycemic control, cardiovascular stability, and the unique challenges posed by COVID-19 and its treatments (Figure 2) (Hebbard et al., 2021). Table 2 summarizing major trials of SGLT2 inhibitors, GLP-1 receptor agonists (RAs), DPP-4 inhibitors, and RAAS inhibitors relevant to diabetes, DCM, and COVID-19.

**TABLE 2:** Major clinical trials of SGLT2 inhibitors, GLP-1 receptor agonists, DPP-4 inhibitors, and RAAS inhibitors in patients with diabetes, diabetic cardiomyopathy, and COVID-19: Cardiovascular and metabolic outcomes.

Drug class	Trial/Study	Population/Setting	Key findings	Study type	Reference
SGLT2 inhibitors	EMPA-REG OUTCOME	Type 2 diabetes with high CV risk	Empagliflozin reduced CV death and all-cause mortality vs. placebo	Randomized, double-blind, placebo-controlled (CV outcomes)	Zinman et al. (2015)
SGLT2 inhibitors	CANVAS Program	Type 2 diabetes with high CV risk	Canagliflozin reduced major CV events but increased risk of amputation at the level of toe or metatarsal	Integrated analysis of two randomized trials (CANVAS Program)	Neal et al. (2017)
SGLT2 inhibitors	DECLARE–TIMI 58	Type 2 diabetes outpatients	Dapagliflozin non-inferior for CV safety; reduced hospitalization for heart failure	Randomized, double-blind, placebo-controlled (CV outcomes)	Wiviott et al. (2019)
SGLT2 inhibitors	DAPA-HF	Patients with heart failure and reduced EF (with/without T2D)	Dapagliflozin reduced composite of worsening HF or CV death	Randomized, double-blind, placebo-controlled (heart failure)	McMurray et al. (2019)
SGLT2 inhibitors	EMPEROR-Reduced	Heart failure with reduced EF (with/without diabetes)	Empagliflozin reduced risk of CV death or HF hospitalization	Randomized, double-blind, placebo-controlled (heart failure)	Packer et al. (2020)
SGLT2 inhibitors	DAPA-CKD	Patients with chronic kidney disease (with/without T2D)	Dapagliflozin reduced risk of kidney failure and CV death or hospitalization	Randomized, double-blind, placebo-controlled (renal outcomes)	Heerspink et al. (2020)
SGLT2 inhibitors (COVID)	DARE-19 (dapagliflozin)	Hospitalized COVID-19 patients with cardiometabolic risk factors	Dapagliflozin safe and well tolerated but did not meet primary endpoints for organ dysfunction/death prevention	Randomized, double-blind, placebo-controlled phase 3	Kosiborod et al. (2021)
SGLT2 inhibitors (COVID)	ACTIV-4a (platform trial)	Hospitalized COVID-19 patients	SGLT2 inhibitors did not improve days free of organ support or mortality vs. usual care; generally, well tolerated	Pragmatic, multicentre, open-label, randomized platform trial	Kosiborod et al. (2024)
SGLT2 inhibitors	Prospective meta-analysis (hospitalized COVID-19)	Hospitalized COVID-19 (pooled RCTs)	Pooled RCT data: safe but no consistent clinically relevant benefit for routine use in hospitalized COVID-19	Prospective aggregate-data meta-analysis of randomized trials	Vale et al. (2024)
GLP-1 receptor agonists	LEADER (liraglutide)	Type 2 diabetes with high CV risk	Liraglutide reduced major adverse cardiovascular events and all-cause mortality	Randomized, double-blind, placebo-controlled (CV outcomes)	Marso et al. (2016b)
GLP-1 receptor agonists	SUSTAIN-6 (semaglutide)	Type 2 diabetes with high CV risk	Semaglutide reduced major adverse CV events (nonfatal MI, stroke, CV death) vs. placebo	Randomized, double-blind, placebo-controlled (CV outcomes)	Marso et al. (2016a)
GLP-1 receptor agonists	REWIND (dulaglutide)	Type 2 diabetes (primary prevention majority)	Dulaglutide reduced major adverse CV events in a broad population including lower baseline CV risk	Randomized, double-blind, placebo-controlled (CV outcomes)	Gerstein et al. (2019)
GLP-1 receptor agonists	EXSCEL (exenatide)	Type 2 diabetes with/without prior CV disease	Once-weekly exenatide noninferior for CV safety; borderline effects on CV outcomes	Randomized, double-blind, placebo-controlled (CV outcomes)	Holman et al. (2017)
GLP-1 receptor agonists (COVID observational)	Comparable outcomes cohort	Patients with diabetes who tested positive for SARS-CoV-2	Current use of GLP-1 RAs not associated with worse COVID-19 outcomes; some analyses suggest neutral or beneficial associations	Observational cohort/registry	Israelsen et al. (2021)
DPP-4 inhibitors	Sitagliptin retrospective multicenter cohort (Italy)	Hospitalized patients with T2D and COVID-19	Sitagliptin use at hospitalization associated with lower mortality in observational matched cohort; requires randomized confirmation	Retrospective multicenter observational study	Solerte et al. (2020)
DPP-4 inhibitors (meta-analyses)	Systematic reviews/meta-analyses	Patients with diabetes and COVID-19 (various cohorts)	Meta-analyses show mixed/neutral effects of DPP-4 inhibitors on COVID-19 mortality and severe outcomes; heterogeneity across studies	Systematic reviews and meta-analyses of observational studies	Hariyanto and Kurniawan (2021)
RAAS inhibitors	HOPE (ramipril)	High-risk patients without heart failure	Ramipril reduced rates of death, MI, and stroke in high-risk patients	Randomized, double-blind, placebo-controlled (primary prevention/high-risk)	Yusuf et al. (2000)
RAAS inhibitors	ONTARGET (telmisartan vs. ramipril)	Patients with vascular disease or high-risk diabetes	Telmisartan noninferior to ramipril; combination increased adverse events without extra benefit	Randomized, double-blind, controlled trial	Yusuf et al. (2008)
RAAS inhibitors	VALIANT (valsartan vs. captopril after MI)	Patients after acute MI with LV dysfunction or heart failure	Valsartan noninferior to captopril for survival after MI; combination not superior	Randomized, double-blind, controlled trial	Pfeffer et al. (2003)
RAAS inhibitors (COVID)	Effects of renin–angiotensin system blockers on outcomes from COVID-19	COVID-19 patients (RCT meta-analyses/observational)	RCT evidence: continuing ACEi/ARB is safe; initiating RAAS blockers not shown to improve COVID-19 outcomes. Observational data mixed	Systematic reviews and meta-analyses	Lee et al. (2024)

### 6.1 Glycemic management

Strict glucose regulation remains a cornerstone of care, with optimal targets of 72–144 mg/dL for plasma glucose and an HbA1c below 7% (Bornstein et al., 2020). Poor glycemic control not only worsens cardiac outcomes but also heightens susceptibility to severe COVID-19. Insulin-dependent patients, especially those with fluctuating glucose levels, should self-monitor at least four times daily—upon waking, before lunch, prior to dinner, and at bedtime (Banerjee et al., 2020).

Metformin remains first-line therapy due to its ability to enhance peripheral uptake, suppress hepatic glucose production, and potentially exert anti-inflammatory effects, while also providing cardiovascular safety. However, caution is warranted in patients with severe infection, renal impairment, or risk of lactic acidosis.

SGLT2 inhibitors (e.g., empagliflozin, dapagliflozin, canagliflozin) offer additional cardiometabolic benefits by promoting renal glucose excretion, inducing natriuresis, and reducing cardiac preload and afterload (Lopaschuk and Verma, 2020). They have demonstrated superior efficacy in preventing heart failure hospitalization compared with GLP-1 receptor agonists (Rolek et al., 2023), although monitoring for dehydration, euglycemic ketoacidosis, and genitourinary infections is essential during acute illness. GLP-1 receptor agonists (liraglutide, semaglutide, dulaglutide) complement these benefits by enhancing glucose-dependent insulin secretion, suppressing glucagon, slowing gastric emptying, and reducing major adverse cardiovascular events (MACE) (Liu, 2024). Their weight-reducing and anti-inflammatory effects make them especially useful for high-risk patients, though gastrointestinal intolerance and rare cases of pancreatitis remain limitations. Combination therapy with SGLT2 inhibitors can further augment cardiometabolic outcomes.

### 6.2 Experimental therapeutics in diabetes and COVID-19

Meng et al. investigated the impact of SARS-CoV-2 (Delta variant) infection in a double-transgenic db/db and K18-hACE2 mouse model of diabetes (Meng et al., 2025). Infected diabetic mice exhibited severe multi-organ injury, higher mortality, pancreatic islet damage, insulin resistance, and dysregulated metabolic hormones, alongside elevated pro-inflammatory cytokines and fibrinolytic activity. Sitagliptin treatment ameliorated hyperglycemia, improved insulin sensitivity and GLP-1 levels, and reduced inflammation and organ injury. Mechanistic analyses implicated hACE2 expression, NF-κB activation, and IRS-1 signaling in disease progression and therapeutic response (Meng et al., 2025). These findings highlight sitagliptin as a potential therapy for mitigating COVID-19 severity in diabetes by targeting metabolic and inflammatory pathways.

### 6.3 Cardiovascular therapeutics

ACE inhibitors, angiotensin receptor blockers (ARBs), and emerging ACE2/Ang-(1–7)–targeted strategies aim to restore RAAS balance in diabetic patients at risk of COVID-19–related cardiac complications. While direct ACE2 blockade may prevent viral entry, it risks impairing ACE2’s protective enzymatic activity. Targeting the AT_1_ receptor provides a safer alternative, reducing Ang II–driven injury while potentially limiting SARS-CoV-2 uptake without compromising ACE2 function (Offringa et al., 2020).

Concurrently, established cardioprotective agents—including ACE inhibitors, ARBs, and β-blockers—remain essential for managing heart failure and preventing arrhythmias during the pandemic, unless contraindicated. Statins provide additional benefit by mitigating vascular inflammation and potentially attenuating COVID-19–related endothelial injury. Given the heightened thrombotic risk in COVID-19, prophylactic anticoagulation is recommended for hospitalized patients, with dosing individualized based on clotting and bleeding risk (Huang et al., 2025; Steenblock et al., 2021).

### 6.4 Immunomodulatory and antiviral therapy

Early antiviral treatment can suppress viral replication and reduce disease severity (Gudima et al., 2023; Singh and de Wit, 2022). IL-6 and IL-1 inhibitors may temper cytokine-driven inflammation, lowering myocardial injury and heart failure risk, although their success depends on individual inflammatory profiles (Mathur and Kottilil, 2022). Corticosteroids, while effective in controlling hyperinflammation in severe COVID-19, can exacerbate hyperglycemia, hypertension, dyslipidemia, and cardiac strain in DCM patients. Therefore, the lowest effective dose should be administered for the shortest duration, with close monitoring of glucose and cardiovascular parameters (Jin and Hu, 2021; Lim et al., 2021).

Emerging evidence also indicates that SARS-CoV-2 infection may precipitate new-onset diabetes, particularly in unvaccinated individuals, by impairing β-cell function, triggering inflammation, and promoting IR. COVID-19 vaccination is therefore strongly recommended for individuals with diabetes or DCM, as the benefits in reducing severe infection and cardiovascular complications outweigh rare risks, such as transient hyperglycemia or mild myocarditis (Huang et al., 2025).

### 6.5 Nanomedicine approaches

Nanomedicine has emerged as a versatile tool for combating COVID-19 and managing comorbid conditions like DCM. Lipid nanoparticles (LNPs) have been central to mRNA vaccines, protecting fragile mRNA, enabling cellular uptake, and eliciting robust immune responses (Pardi et al., 2018; Swetha et al., 2023). Beyond vaccines, polymeric, gold, dendrimer, and silica-based nanoparticles enhance targeted delivery of antiviral drugs, improve pharmacokinetics, reduce off-target effects, and can serve as viral decoys or rapid diagnostics (Mitchell et al., 2021; Patra et al., 2018).

In DCM, nanocarriers improve the bioavailability and tissue-specific delivery of cardioprotective, anti-inflammatory, and antidiabetic agents, minimizing systemic toxicity and allowing controlled release in metabolically compromised hearts (Choudhury et al., 2024a; He et al., 2021). PLGA-based nanosystems have improved endothelial function, ventricular performance, and eNOS/VEGF-A expression in DCM models, attenuating vascular damage and remodeling (Luyan, 2018). Similarly, acid fibroblast growth factor (aFGF)-loaded nanoliposomes combined with ultrasound-targeted microbubble destruction (UTMD) reduced cardiac apoptosis and fibrosis, increased microvascular density, and restored systolic and diastolic function (Zhang et al., 2017). Nano-curcumin (≈200 nm) has also demonstrated anti-fibrotic, antioxidant, and anti-AGEs effects in diabetic hearts (Abdel-Mageid et al., 2018). Finally, metal nanoparticles such as ZnO and gold show cardioprotective effects at controlled doses by reducing oxidative markers and apoptosis, though care must be taken to avoid toxicity at higher concentrations (Liu et al., 2022). Overall, while nanomedicine offers innovative avenues for precision therapy in cardiometabolic disease and COVID-19, its current application remains largely preclinical, and further rigorous clinical investigation is needed before these strategies can be integrated into standard care.

## 7 Conclusion and future directions

DCM in the setting of COVID-19 represents a convergence of metabolic dysregulation, chronic inflammation, RAAS imbalance, endothelial dysfunction, and thrombotic risk, collectively exacerbating myocardial injury and long-term cardiovascular complications. Effective management requires a personalized approach that integrates antidiabetic therapies with cardiovascular protection, while carefully controlling blood pressure, lipids, and coagulation. Interdisciplinary care informed by mechanistic insights enables targeted interventions addressing metabolic, inflammatory, and hemodynamic pathways, ultimately reducing morbidity and mortality in this high-risk population.

Looking forward, key research questions include: What are the relative contributions of endothelial injury, microvascular thrombosis, and impaired coronary flow reserve to acute and chronic cardiac dysfunction in DCM patients with COVID-19? How does chronic metabolic inflammation in diabetes interact with COVID-19–induced cytokine responses to drive cardiac remodeling, fibrosis, and arrhythmogenic risk? Can patient-specific molecular or immunometabolic profiles predict responsiveness to SGLT2 inhibitors, GLP-1 receptor agonists, RAAS modulators, or anti-inflammatory therapies during COVID-19? To what extent does COVID-19 accelerate the progression of DCM, and are there identifiable windows for intervention to prevent persistent myocardial injury, heart failure, or sudden cardiac death? Addressing these questions will be crucial for developing precision therapies and improving outcomes for patients at the intersection of diabetes, DCM, and COVID-19.

## Glossary

ACE2: Angiotensin-Converting Enzyme 2
AGEs: Advanced Glycation End Products
AMPK: AMP-Activated Protein Kinase
ARB: Angiotensin Receptor Blocker
ARDS: Acute Respiratory Distress Syndrome
CaMKKβ: Calcium/Calmodulin-Dependent Protein Kinase Kinase β
CD36: Cluster of Differentiation 36
CRP: C-Reactive Protein
CTS: Cytokine Storm
DCM: Diabetic Cardiomyopathy
DM: Diabetes Mellitus
eNOS: Endothelial Nitric Oxide Synthase
ER: Endoplasmic Reticulum
FGF21: Fibroblast Growth Factor 21
FFAs: Free Fatty Acids
O-GlcNAc: O linked N-Acetylglucosamine
ICAM-1: Intercellular Adhesion Molecule 1
IL-6: Interleukin-6
IR: Insulin Resistance
JNK: c-Jun N-terminal Kinase
LDH: Lactate Dehydrogenase
LKB1: Liver Kinase B1
MACE: Major Adverse Cardiovascular Events
MAPK: Mitogen-Activated Protein Kinase
MD2: Myeloid Differentiation Protein 2
NETs: Neutrophil Extracellular Traps
NFκB: Nuclear Factor Kappa-light-chain-enhancer of Activated B Cells
NO: Nitric Oxide
ORs: Odds Ratios
PAI-1: Plasminogen Activator Inhibitor-1
PKC: Protein Kinase C
RAAS: Renin–Angiotensin–Aldosterone System
RAGE: Receptor for AGE
ROS: Reactive Oxygen Species
RT-PCR: Reverse Transcriptase Polymerase Chain Reaction
SARS-CoV-2: Severe Acute Respiratory Syndrome Coronavirus 2
SGLT2: Sodium–Glucose Cotransporter 2
T1D: Type 1 Diabetes
t-PA: Tissue Plasminogen Activator
TF: Tissue Factor
TMPRSS2: Transmembrane Serine Protease 2
TNF-α: Tumor Necrosis Factor-Alpha
UTMD: Ultrasound-Targeted Microbubble Destruction
VCAM-1: Vascular Cell Adhesion Molecule 1
